# Mechanochemical synchronization of force oscillations in connective tissue models

**DOI:** 10.64898/2026.10.18.759356

**Published:** 2026-10-22

**Authors:** David Böhringer, Leah Vogler, Christoph Mark, Pamela L. Strissel, Jonas Röder, Delf Kah, Lovis Schween, Olivier Zajac, Annamaria Härtl, Lena Fischer, Silvia Budday, Richard Gerum, Harald Herrmann, Lilli Winter, Gerhard Wiche, Wolfgang H. Goldmann, Giovanni Cappello, Danijela Matic Vignjevic, Martial Balland, Aldo R. Boccaccini, Reiner Strick, Ingo Thievessen, Thomas Boudou, Ben Fabry

## Abstract

Cells in a tissue coordinate their behavior collectively by mechanical and chemical feedback with their surroundings. To study how such feedback mechanisms interact, we embed multicellular spheroids from mouse and primary human fibroblasts of different origin in 3D collagen matrices. Immediately after embedding, spheroids apply steadily increasing, radially symmetric, inward-directed forces to the surrounding matrix. Typically, within a few hours, collective force oscillations emerge with an average time interval of *≈* 30 min. Similar force oscillations also arise in engineered, parallel-aligned microtissues attached to flexible pillars. Force oscillations propagate between neighboring cells as a mechanical wave, accompanied by calcium waves that travel at comparable speeds of several hundred µm/min. Blocking gap junctions or mechanosensitive Piezo1 channels impairs these oscillations, indicating that calcium signaling contributes to their propagation. Their amplitude and frequency are regulated by integrin-mediated feedback in actin-rich protrusions and by ERK signaling, which also controls collective invasion into the surrounding matrix. Neighboring spheroids mechanically synchronize their force oscillations in phase and frequency over distances of up to *≈* 2 mm without direct contact. Together, our results show that oscillatory mechanochemical feedback enables multicellular systems to coordinate force generation and invasive migration across cellular and millimeter length scales in 3D matrices.

